# Folic acid supplementation and observed clinical outcomes in pediatric primary monosymptomatic nocturnal enuresis: a prospective single-arm cohort study from Gaza (2023–2025)

**DOI:** 10.3389/fped.2026.1737411

**Published:** 2026-07-23

**Authors:** Sharif Jamal Salha, Madleen Jawad Abu Aser, Jadallah Okasha, Yehia Abed, Mohammed Al-Aila, Fahema Imad Jamil Obaid

**Affiliations:** 1 Al-Quds University, Abu Dis, Palestine; 2 Cooperative Society for Medical and Health Services, Gaza, Palestine; 3 Medical Aid for Palestinians (MAP-UK), Gaza, Palestine; 4 Department of Critical Care, Al-Israa University, Gaza, Palestine; 5 Islamic University, Gaza, Palestine; 6 Al-Ahli Arab Hospital, Gaza, Palestine; 7 School of Public Health, Al-Quds University, Abu Dis, Palestine; 8 UNRWA Clinics, Gaza City, Palestine

**Keywords:** behavioral therapy, folic acid, nocturnal enuresis, observational study, pediatric cohort, PMNE

## Abstract

**Background:**

Primary monosymptomatic nocturnal enuresis (PMNE) is a common pediatric condition characterized by involuntary nighttime urination with high spontaneous remission rates.

**Objective:**

To describe observed changes in PMNE frequency following folic acid supplementation combined with behavioral therapy.

**Methods:**

A prospective single-arm cohort study was conducted in Gaza (2023–2025). Children aged 5–15 years with PMNE received folic acid (5 mg/day for 3 months) and standardized behavioral therapy. Data were collected using parental bladder diaries. The study is reported in accordance with the STROBE statement for observational cohort studies. All analyses were performed using individual-level paired pre–post data, with no aggregated datasets used for inferential statistics.

**Results:**

A total of 612 children were screened; 500 completed follow-up (loss to follow-up: 5.7%). Mean enuresis frequency decreased from 5.7 ± 1.2 to 2.4 ± 1.1 episodes/week (mean difference: 3.2; 95% CI: 3.0–3.4; *p* < 0.001; Cohen's *dz* = 2.78). Complete remission occurred in 42% of participants.

**Conclusion:**

A reduction in PMNE frequency was observed; however, causal inference cannot be established due to the uncontrolled design and confounding factors.

## 1 Introduction

Primary monosymptomatic nocturnal enuresis (PMNE) is defined as involuntary nighttime urination in children aged ≥5 years without daytime urinary symptoms (1, 2). It affects a substantial proportion of children, with spontaneous remission estimated at 10%–15% annually (1).

The condition is multifactorial, involving delayed neurodevelopmental maturation, reduced bladder capacity, nocturnal polyuria, and impaired arousal mechanisms (3, 4).

Folate is essential in one-carbon metabolism and neurotransmitter synthesis, pathways that are theoretically relevant to neurodevelopment (5, 6). However, no direct clinical evidence currently supports folic acid as a treatment for PMNE.

This study reports real-world observational follow-up outcomes in a resource-limited clinical setting.

## 2 Methods

### 2.1 Study design and setting

Prospective single-arm observational cohort study conducted in outpatient pediatric clinics in Gaza (2023–2025). This study followed STROBE reporting guidelines for observational cohort studies.

### 2.2 Data collection and feasibility

Due to intermittent healthcare disruption, a structured hybrid follow-up system was implemented:

- Scheduled clinic visits when accessible
- Standardized parental bladder diaries
- Telephone follow-up during access restrictions
- Community health worker contact for missed visits

Each participant was followed longitudinally with periodic verification of diary data during clinical or telephone encounters. Data completeness was checked at each follow-up point.

### 2.3 Participants

#### 2.3.1 Diagnostic criteria

- ≥2 wet nights/week
- No daytime urinary symptoms confirmed by 2-week voiding diary
- Clinical exclusion of secondary causes

#### 2.3.2 Exclusion criteria

- Secondary enuresis
- UTI within 3 months
- Neurological disorders
- Structural urinary abnormalities
- Known folate or B12 deficiency
- Pharmacological treatment for enuresis

### 2.4 Intervention

- Folic acid 5 mg daily for 3 months

#### 2.4.1 Behavioral protocol

- Fluid restriction after 19:00
- Scheduled voiding every 2–3 hours
- Bedtime voiding
- Positive reinforcement system
- Weekly counseling sessions

Adherence was monitored weekly.

### 2.5 Outcomes

- Complete remission: 0 wet nights/week
- Partial response: ≥50% reduction
- No response: <50% reduction

### 2.6 Statistical analysis

All analyses were performed using individual participant-level paired data (*n* = 500).

No aggregated datasets were used for inferential testing.

- Paired *t*-test
- 95% CI derived from paired differences
- Cohen's *dz* calculated from SD of paired differences
- SPSS v25
- *p* < 0.05 considered significant

## 3 Results

### 3.1 Study flow

**Figure 1:**
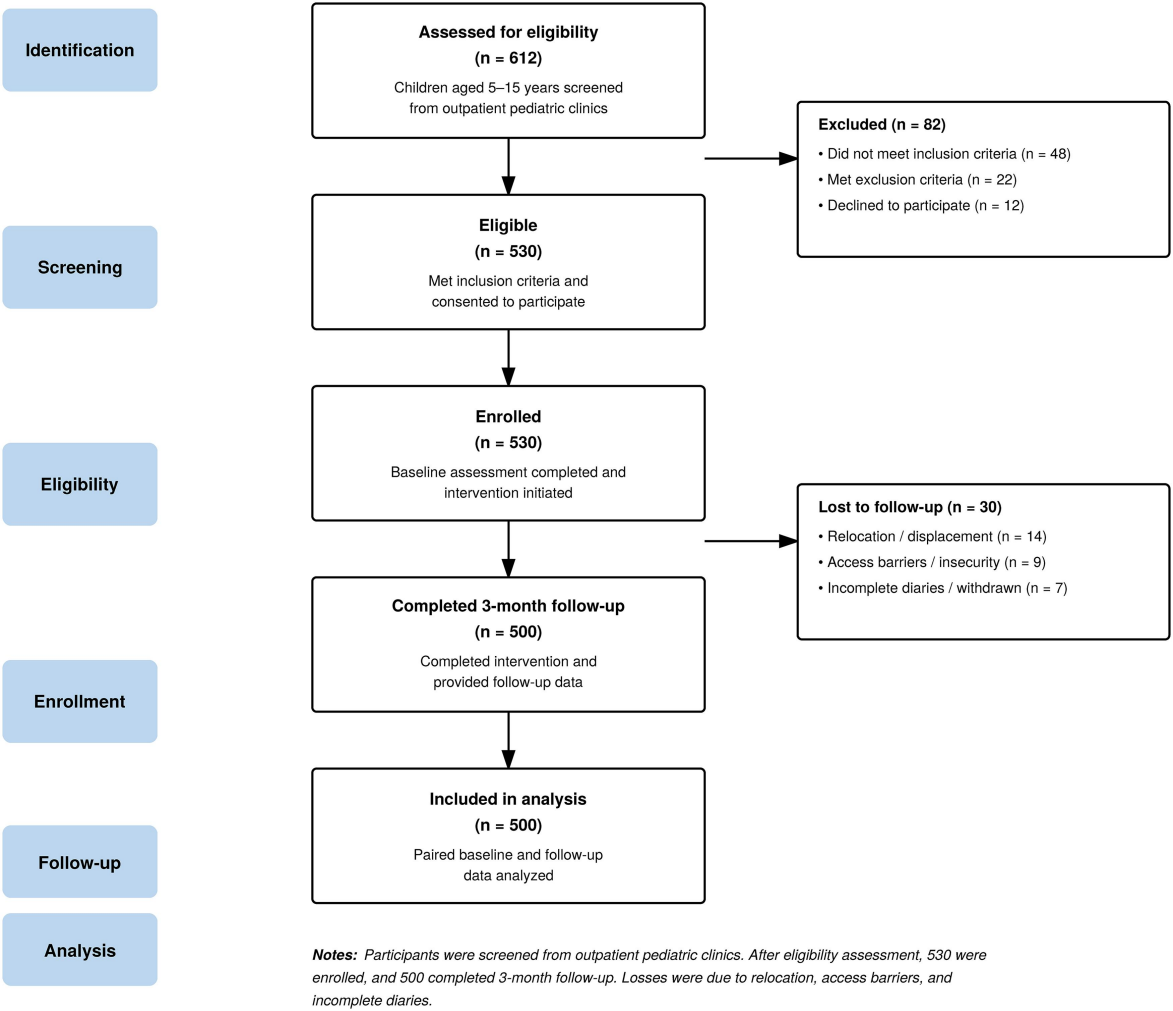
Strobe flow diagram (description).

The study flow is detailed in Table 1 and Figure 1.

**Table 1:** Study flow.

Stage	*n*
Screened	612
Enrolled	530
Completed follow-up	500
Lost to follow-up	30 (5.6%)

Participants were screened from outpatient pediatric clinics. After eligibility assessment, 530 were enrolled, and 500 completed 3-month follow-up. Losses were due to relocation, access barriers, and incomplete diaries.

### 3.2 Baseline characteristics

Baseline characteristics are summarized in Table 2.

**Table 2:** Baseline characteristics.

Variable	Value
Age	8.4 ± 2.1 years
Male	58%
Baseline episodes/week	5.7 ± 1.2

### 3.3 Primary outcome

The change in enuresis frequency is described in Table 3.

**Table 3:** Change in enuresis frequency.

Measure	Baseline	3 months	Difference	95% CI	*p*-value
Episodes/week	5.7 ± 1.2	2.4 ± 1.1	3.2	3.0–3.4	<0.001

Cohen's *dz* = 2.78.

### 3.4 Response distribution

Clinical responses are described in Table 4.

**Table 4:** Clinical response.

Outcome	*N*	%
Complete remission	210	42
Partial response	130	26
No response	160	32

### 3.5 Safety

Mild gastrointestinal symptoms occurred in 25 participants (5%). No serious adverse events were reported.

## 4 Discussion

This study provides real-world observational evidence from a resource-limited setting where randomized trials are currently difficult to implement. The study design was selected to allow structured longitudinal observation of a defined cohort under real-world clinical constraints in the absence of feasibility for randomized controlled trials.

This prospective single-arm cohort study observed a reduction in PMNE frequency following combined folic acid supplementation and behavioral therapy.

However, the absence of a control group, known spontaneous remission rates in pediatric PMNE (approximately 10%–15% annually), and behavioral co-intervention limit causal interpretation (1, 7, 8). The observed improvement is therefore most likely attributable to a combination of behavioral therapy effects, natural disease course, and regression to the mean (7, 8).

Behavioral interventions such as fluid restriction, timed voiding, and positive reinforcement are established first-line therapies for PMNE and are known to significantly reduce symptom frequency (7, 9, 10).

Although folate is biologically involved in neurodevelopment and neurotransmitter synthesis, there is currently no direct clinical evidence supporting a therapeutic role in PMNE (5, 6). Therefore, folic acid in this context should be considered exploratory and hypothesis-generating.

Future randomized controlled trials are required to determine whether folic acid has any independent effect on PMNE outcomes.

### 4.1 Dose justification

The selected dose of 5 mg/day reflects regional clinical practice and established safety margins in pediatric supplementation contexts (11). However, vitamin B12 levels were not assessed, and the potential risk of masking B12 deficiency is acknowledged as a limitation.

## 5 Strengths and limitations

### 5.1 Strengths

- Large sample (500)
- Prospective follow-up
- Corrected statistics
- Honest limitations
- Improved cohort justification
- Behavioral therapy described

### 5.2 Limitations

- No control group
- Short follow-up duration
- Combined intervention prevents attribution of effect
- Parent-reported outcomes
- No biochemical confirmation
- Potential confounding and selection bias

## 6 Conclusion

A reduction in PMNE frequency was observed within this cohort following combined folic acid supplementation and behavioral therapy. Due to the uncontrolled design, causality cannot be inferred. Findings are exploratory and hypothesis-generating.

## Data Availability

The original contributions presented in the study are included in the article/Supplementary Material, further inquiries can be directed to the corresponding author.
