# Supplementary material for: Folic acid supplementation and observed clinical outcomes in pediatric primary monosymptomatic nocturnal enuresis: a prospective single-arm cohort study from Gaza (2023–2025)
Source: Front Pediatr. 2026 Jul 23;14:1737411. doi: 10.3389/fped.2026.1737411 (PMC13443297; doi:10.3389/fped.2026.1737411)
Supplement: Supplementary file 1 [file Supplementaryfile1.docx]

# 3. Results

## 3.1 Baseline Characteristics (Table 1)

Table 1. Baseline Characteristics

- Sample size: 500
- Mean age: 8.4 ± 2.1 years
- Male: 58%
- Female: 42%
- Mean baseline episodes/week: 5.6 ± 1.2

## 3.2 Clinical Outcomes (Table 2)

Table 2. Pre–Post Comparison

- Baseline mean: 5.6 ± 1.2
- Post-treatment mean: 2.4 ± 1.1
- Mean difference: 3.2
- 95% CI: 3.0–3.4
- t-value: 41.8
- p<0.001
- Cohen’s d=2.2

## 3.3 Response Distribution (Table 3)

Table 3. Treatment Response

- Complete remission: 42%
- Partial response: 26%
- No response: 32%

- Table 1. Baseline Characteristics of Study Participants (n = 500)

| **Characteristic** | **Value** |
| --- | --- |
| **Sample size** | 500 |
| **Mean age (years)** | 8.4 ± 2.1 |
| **Sex, n (%)** |  |
| **Male** | 290 (58%) |
| **Female** | 210 (42%) |
| **Mean baseline episodes/week** | 5.6 ± 1.2 |

- **3.2 Clinical Outcomes (Pre–Post Comparison)**
- **Table 2. Pre–Post Treatment Outcomes**

| **Outcome**  **Measure** | **Baseline**  **Mean ± SD** | **Posttreatment Mean ± SD** | **Mean**  **Difference**  **(95% CI)** | **t-**  **value** | **p-value** | **Effect Size**  **(Cohen’s d)** |
| --- | --- | --- | --- | --- | --- | --- |
| **Episodes per week** | 5.6 ± 1.2 | 2.4 ± 1.1 | 3.2 (3.0–3.4) | 41.8 | <0.001 | 2.2 |

- *Interpretation:* The intervention significantly reduced the weekly episodes, with a very large effect size (Cohen’s d = 2.2), indicating a strong clinical impact.

- **3.3 Response Distribution**
- Table 3. Treatment Response Among Participants

| **Response Category** | **n (%)** |
| --- | --- |
| **Complete remission** | 210 (42%) |
| **Partial response** | 130 (26%) |
| **No response** | 160 (32%) |
